# An Acute Limb Ischemia Concomitant With a Myocardial Infarction

**DOI:** 10.7759/cureus.13538

**Published:** 2021-02-24

**Authors:** Darar Charmake, Ismahane Lahmidi, Mohamed Boutaybi, Noha Elouafi

**Affiliations:** 1 Cardiology, Mohammed I University/Mohammed VI University Hospital, Oujda, MAR; 2 Cardiology, Mohammed I University, Epidemiological Laboratory of Clinical Research and Public Health, Mohammed VI University Hospital, Oujda, MAR

**Keywords:** acute limb ischemia, myocardial infarction, revascularization

## Abstract

Acute limb ischemia (ALI) is an abrupt interruption of limb blood flow due to acute occlusion of the peripheral artery. Its concomitant occurrence with myocardial infarction (MI) constitutes a rare but serious clinical situation that worsens the functional prognosis of the affected limb or leads to the death of the patient.

We report a case of an 87-year-old male patient who was diagnosed with acute left lower limb ischemia concomitant with MI. The diagnosis was based on clinical, electrical data and arterial angiography scan of limb findings. Thanks to urgent myocardial revascularization associated with that of the lower limb, curative heparin therapy, and armed clinical surveillance, the evolution was favorable.

## Introduction

Acute limb ischemia (ALI) is defined as any sudden decrease in limb perfusion, which threatens both limb viability and mortality [1]. It is a serious disease, i.e., a potentially critical clinical condition in patients with multiple comorbidities. Among the various causes of ALI are thromboembolism, usually of cardiac origin (atrial fibrillation, history of myocardial infarction [MI]), in situ thrombosis, trauma, and peripheral aneurysms [2,3]. However, ALI with concomitant MI is extremely rare and/or rarely reported as warranting a strategy of rapid revascularization. We report a case of acute left lower limb ischemia concomitant with an inferior wall MI successfully treated by primary angioplasty and urgent revascularization of the lower limb.

## Case presentation

We report the case of an 87-year-old patient who has, as risk factors, diabetes treated with insulin and hypertension. He was admitted to the emergency department for an atypical chest pain, vomiting, and sweating, which appeared four hours before his admission.

**Figure 1:**
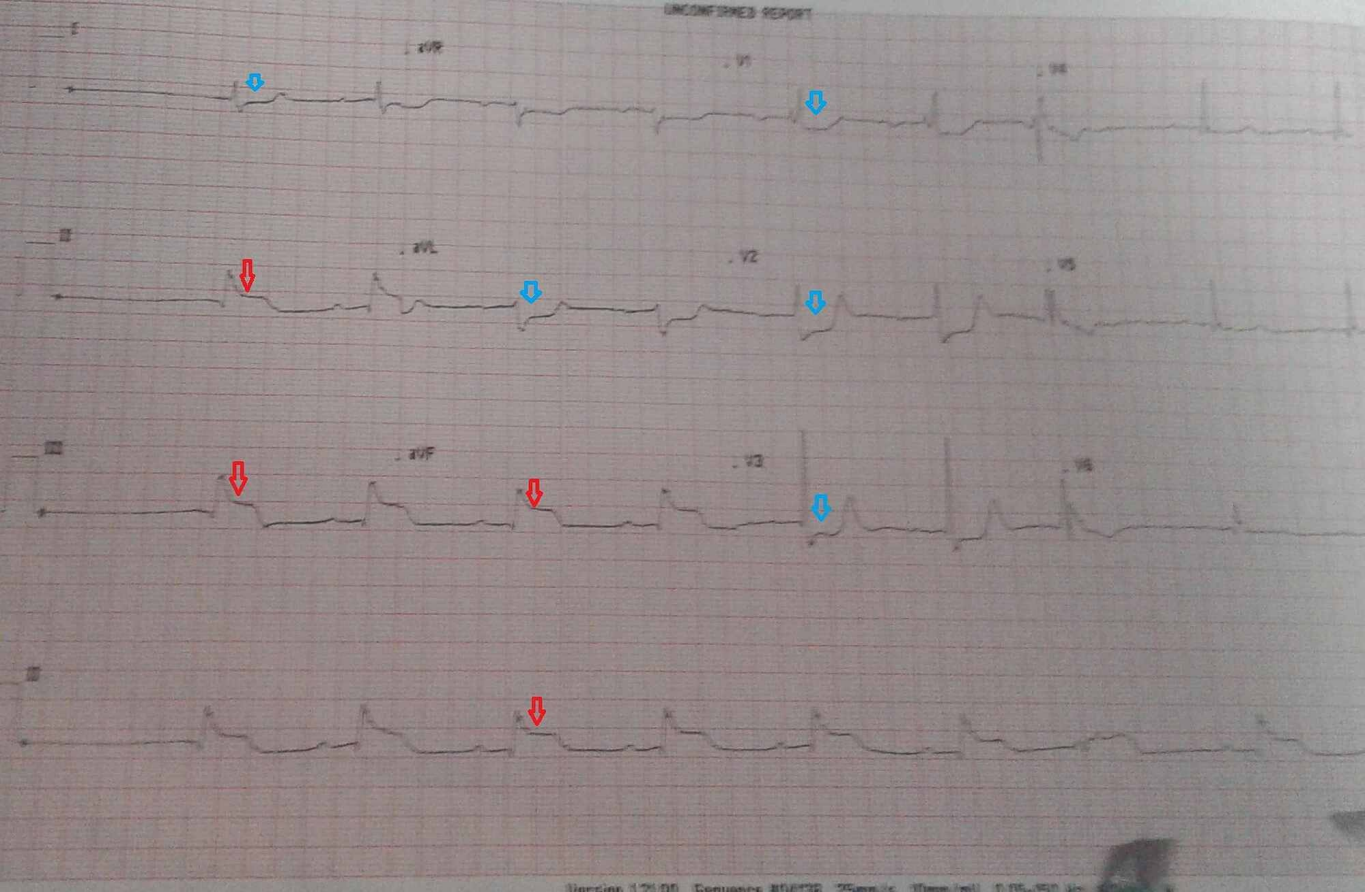
Electrocardiogram on presentation showing ST-segment elevations in leads II, III, and aVF (red arrows) with reciprocal changes in leads I, aVL, and V1-V3 (blue arrows) aVF, Augmented Vector Foot; aVL, augmented Vector Left.

The clinical examination at admission revealed a conscious patient with blood pressure at 100/50 mmHg, heart rate at 54/min, SpO2 (oxygen saturation) at 98%, and the abolition of the pedal and posterior tibial pulses. Popliteal and femoral pulses were also present. The electrocardiogram revealed ST-segment elevation in the inferior leads and Q waves of necrosis without negative T waves (Figure 1).

The laboratory assessment showed a severe microcytic hypochromic anemia (hemoglobin at 6.8 g/dl), moderate renal failure (clearance by modification of diet in renal disease [MDRD] at 33 ml/min), and a normal level of C-reactive protein. Echocardiography objectified a left ventricular (LV) ejection fraction at 55% with inferior wall motion abnormalities and a right ventricular (RV) systolic dysfunction.

**Figure 2:**
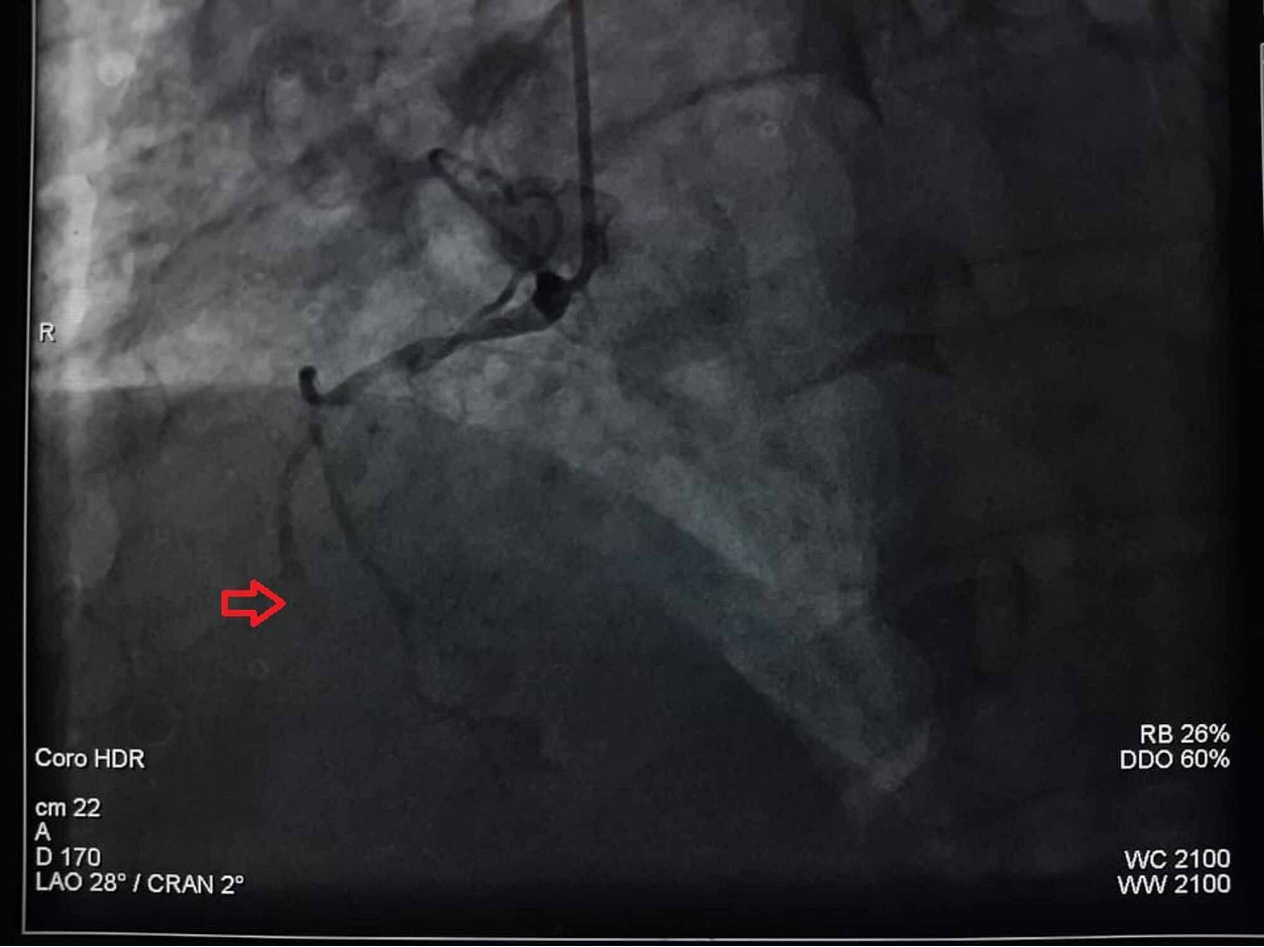
Coronary angiography showing the middle right coronary artery (RCA) occlusion (red arrow)

Once the diagnosis of inferior ST-elevation myocardial infarction (STEMI) with persisting chest pain was made, the patient was immediately taken to the cardiac catheterization laboratory for primary angioplasty. The coronarography revealed an occlusion of the middle right coronary artery (RCA), and the patient underwent angioplasty of the right coronary with an active stent (Figure 2).

**Figure 3:**
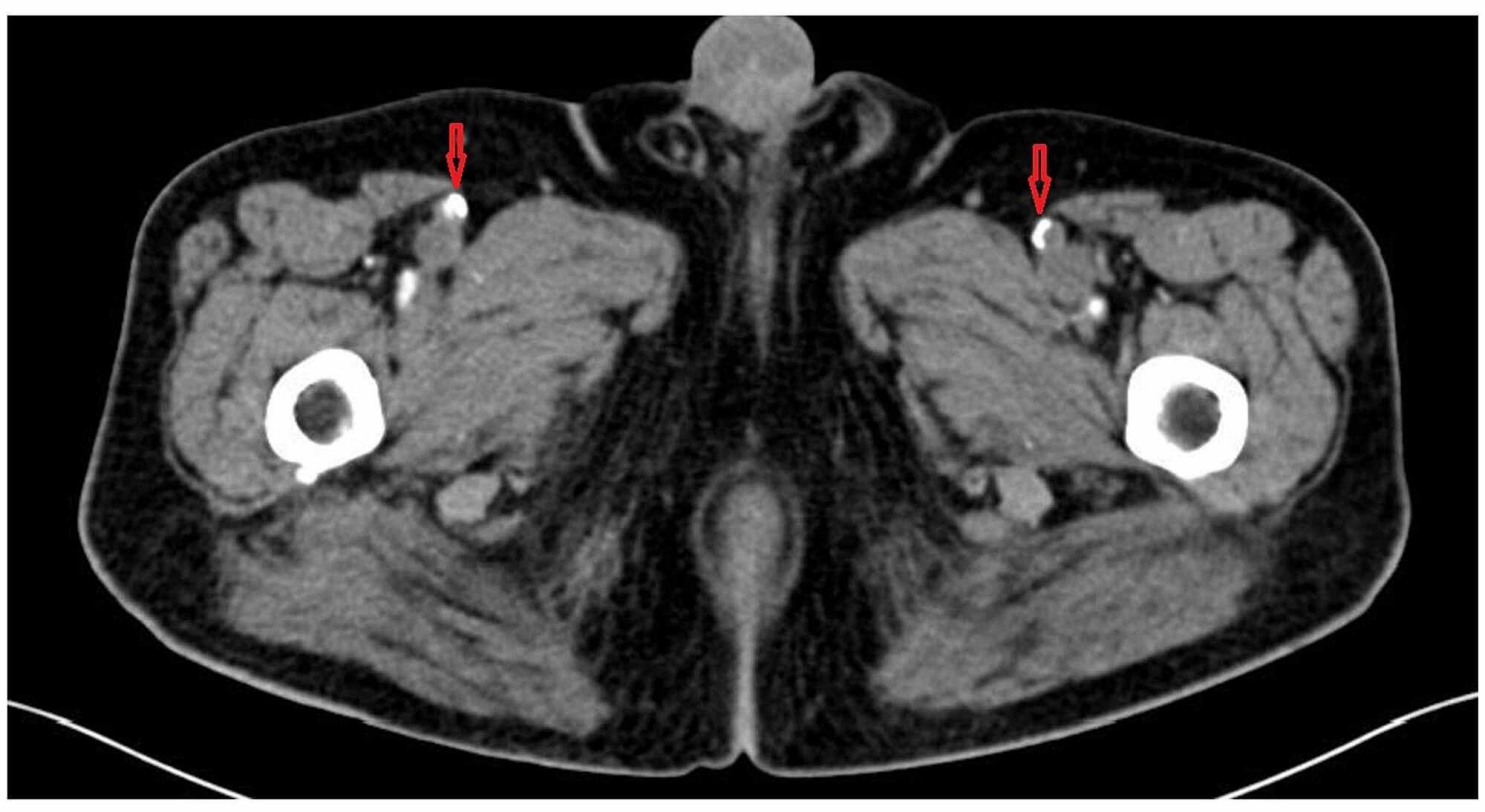
Computed tomography angiogram (CTA) of lower extremities showing acute vascular occlusion at the level of left superficial femoral artery (red arrow).

In the postangioplasty evolution, the patient presented a brutal and intense pain of the left lower limb, becoming cold and hypoesthesic, thus making the diagnosis of ALI a suspect. A computed tomography (CT) angiography (Figure 3) was realized, which revealed an acute vascular occlusion at the level of left superficial femoral artery. Therefore, we have performed an open thrombectomy of the responsible artery thrombus associated with an anticoagulant treatment. The postoperative outcome was marked by the restoration of blood flow in the femoral and downstream vascular area demonstrated by the appearance of palpable distal pulses.

During the course of hospitalization, the patient progressed with hemodynamics and recovery of ischemic process, which allowed discharge with medical treatment according to current recommendations.

## Discussion

ALI is a life-threatening medico-surgical emergency, an important cause of morbidity and mortality, causing limb amputation in 5%-12% in 30 days and mortality in 10%-38% [4]. It may be due to a heart cause, for example, left atrial thrombus secondary to atrial fibrillation or LV thrombus following a MI [2,3]. The other mechanism is in situ thrombosis, frequently observed in patients with underlying obliterating arteriosclerosis.

ALI and MI have similar mechanisms. Acute thrombotic arterial occlusion causes ischemia and tissue necrosis without rapid revascularization. Although these two entities may be due to an identical pathophysiological mechanism, their simultaneous appearance is extremely rare. Our patient did not have typical angina pain, and acute MI was purely suspected in the presence of atypical pain that appeared four hours earlier with vomiting and ST-segment elevation on the electrocardiogram. The long course of diabetes could explain this symptomatology.

ALI is usually manifested by the sudden onset of limb pain, such as grinding, often accompanied by paresthesia, weakness, or abolition of one or more peripheral pulses [5]. Semiologically identical pain with the abolition of the posterior pedal and tibial pulses was found in our case. Suspected limb occlusion is evaluated using arterial ultrasound, CTA, and magnetic resonance angiography [6,7]. Concerning therapeutic management, our patient was successfully treated by urgent myocardial revascularization of the culprit artery, curative dose of heparin therapy, and thrombectomy of the responsible artery thrombus.

Various options can be proposed for emergency revascularization of ALI (Fogarty embolectomy, bypass, in situ thrombolysis, or even limb amputation) depending on the severity of ischemia based on the Rutherford classification. Indeed, in situ thrombolysis is the treatment of choice if the ischemia is mild (grade I or II A), unlike the severe ischemia (grade II B and III of Rutherford), which justifies open revascularization with possible percutaneous manual thrombo-aspiration [1].

However, surgical treatment is weighted with a high mortality rate of around 15%-25% in 30 days [8]. Randomized studies have shown that thrombolysis is generally as effective as surgery in appropriately selected patients [8]. Fogarty's mechanical thrombectomy has a high success rate with low amputation and mortality rates [9]. Due to the high rate of morbidity and mortality in this pathology, the presence of comorbidities (diabetes, hypertension, deep anemia), the potential risk of devascularization syndromes such as metabolic disorders (hyperkalemic, metabolic acidosis) and acute renal failure, and regular clinical and biological monitoring are essential [10]. This complication is grafted with a mortality of 30%-80% due to the release into the systemic circulation of various metabolites causing multi-organ failure [10]. Only an attitude based on rapid management and strict clinical and biological monitoring would improve the prognosis both in terms of coronary ischemia and ALI.

## Conclusions

This observation makes it possible to conclude that acute ischemia of the lower limb occurring concomitantly with a MI is a rare but serious clinical condition, and the atherosclerotic etiology by the formation of thrombosis which it causes is responsible for it. A meticulous clinical evaluation supplemented in case of doubt by a CT scan is essential in order to implement an adequate therapeutic strategy aimed at preserving the vital and functional prognosis of the concerned member.
